# Nanoparticles of Bioactive Metals/Metal Oxides and Their Nanocomposites with Antibacterial Drugs for Biomedical Applications

**DOI:** 10.3390/ma15103602

**Published:** 2022-05-18

**Authors:** Tatyana Shabatina, Olga Vernaya, Aleksei Shumilkin, Alexander Semenov, Mikhail Melnikov

**Affiliations:** 1 Department of Chemistry, M.V. Lomonosov Moscow State University, Moscow 119991, Russia; olga_vernaya@mail.ru (O.V.); alexpard99@gmail.com (A.S.); alexandrm.semenov@mail.ru (A.S.); melnikov46@mail.ru (M.M.); 2 Department of Natural Sciences, N.E. Bauman Moscow State Technical University, Moscow 105005, Russia; 3 Department of Biology, M.V. Lomonosov Moscow State University, Moscow 119991, Russia

**Keywords:** nanometals, metal oxides nanoparticles, antibiotics, resistant bacteria strains, hybrid nanocomposites, antibacterial activity

## Abstract

The increasing appearance of new strains of microorganisms resistant to the action of existing antibiotics is a modern problem that requires urgent decision. A promising potential solution is the use of nanoparticles of bioactive metals and their oxides as new antibacterial agents, since they are capable of affecting pathogenic microorganisms by mechanisms different from the mechanisms of action of antibiotics. Inorganic nanoparticles possess a wide spectrum of antibacterial activity. These particles can be easily conjugated with drug molecules and become carriers in targeted drug-delivery systems. This paper discusses the benefits and prospects of the application of nanoparticles from metals and metal oxides and their nanocomposites with antibacterial drugs.

## 1. Introduction

Nanotechnology, which deals with the manipulation and fabrication of nanoscale materials (with particles and grains in ranging in size from 1 to 100 nm) can be used in rapidly developing new areas: nanoelectronics, nanophotonics, nanooptics, nanocatalysis and nanoenergetics [1]. It has also opened new horizons in medicine and led to the emergence of its new branch—nanomedicine [2]. Nanoparticles (NPs) and nanosystems (NS) have found numerous biomedical applications over the last decades. A significant contribution to this expansion is made by inorganic nanoparticles (INPs); that is, metals and their oxides along with their hybrid nanocomposites (HNCs) with drug components. They can be considered as carriers, vectors, and active components in targeted drug delivery systems [3,4,5,6]. Magnetic INPs are promising agents for magnetic resonance imaging (MRI) [7,8] and contribute considerably to the development of tissue engineering [9,10]. These INPs are promising agents for magnetic hyperthermia [11,12]. Magnetic separation and biosensors based on INPs simplify laboratory diagnostics [13,14,15].

The introduction of antibiotics in the treatment of bacterial diseases in the mid-20th century revolutionized medicine. A large number of previously incurable diseases became no longer fatal. However, the frequent and unreasonable use of antibiotics has led to the growth and spread of resistant pathogenic strains of microorganisms. The creation of new antibacterial drugs takes years, or even decades, of laboratory research, preclinical and clinical trials. Furthermore, it requires a large amount of time, laboratory equipment and financial resources. Unfortunately, the rate of production of new antibacterial drugs is inadequate to address the rate of emergence and development of resistant strains of microorganisms.

Drug-resistant infections are a major cause of death and have resulted in a serious risk to public health. Additionally, increasing resistance to antimicrobial drugs is emerging as an urgent problem in medicine [16]. In this context, INPs with a bactericidal effect could be considered as a promising solution to this problem [17]. They attack pathogenic microorganisms using various mechanisms that differ from the mechanisms of antibiotics action. First, INPs from metals and metal oxides with a pronounced bactericidal effect, such as gold, silver, zinc oxide, and copper and its oxides are being considered as new antibacterial agents [18,19,20,21,22,23]. INPs in a drug system may have other functions. They are also potential carriers and magnetic vectors in targeted drug delivery in the forms of HNCs and NSs. Therefore, the activity of particles with less pronounced antibacterial activity has also been studied in scientific publications of recent years. Furthermore, there is a trend towards the creation of hybrid nanocomposites (HNCs), which consist of INPs (as active substances and carriers) and drug substances. In such HNCs, INPs serve as a carrier and expand the spectrum of bactericidal action. The establishment of a synergistic increase in antibacterial activity of HNCs in comparison to separate actions of INPs and antibiotic substances alone makes such systems even more attractive [23].

## 2. INPs as Promising Antibacterial Agents

INPs including metals (Au, Ag, Cu, and Fe) and metal oxides (CuO, ZnO, MgO, CaO, TiO_2_, magnetite, and ferrites) have been reported as exhibiting antibacterial potential against both Gram-negative and Gram-positive bacteria strains [20,21,22,23].

### 2.1. Ag INPs

Silver has been known and used as a potent antimicrobial agent since early times. The bactericidal properties of silver were used in ancient Greece and Rome. The earliest therapeutic application of silver was reported in 1500 BC during the Han dynasty in China [24]. Silver INPs have become one of the most widely studied topics in recent decades because of their extraordinary antimicrobial efficiency, even at low concentrations. Their markedly expanded bactericidal behaviors render them potent and novel antimicrobial agents [25,26]. Such enhanced antibacterial activity of Ag at the nanoscale is due to the large surface-to-volume ratio (which increases the release of silver ions and facilitates interaction with the bacterial cells) and direct intra-cellular uptake of Ag NPs, leading to localized release of Ag ions [27]. Unlike silver salts, silver NPs do not contain counter-ions that can affect biological processes. They provide a controlled release of silver ions, which reduces fluctuations in their concentration in the lesion.

Ag INPs effectively inhibit a wide spectrum of Gram-positive and Gram-negative bacteria, such as *Bacillus cereus*, *Staphylococcus aureus*, *Escherichia coli*, *Pseudomonas aeruginosa* [28], *M. luteus* [28], *Streptococcus agalactiae*, *Aeromonas hydrophila*, *Vibrio alginolyticus* [29], *Vibrio natriegens* [28], *Salmonella typhi*, *Vibrio cholerae*, *Enterococcus faecalis*, *Hafnia alvei*, *Acinetobacter baumannii*, *Bacillus subtilis* [30], *Klebsiella pneumoniae*, *Salmonella typhimurium*, *Salmonella enteritidis* [31], *Bacillus megaterium*, *Proteus vulgaris*, *Shigella sonnei* [32], *Listeria monocytogenes*, *Penicilliumitalicum*, *Bacillus subtilis*, *B. subtilis*, *S. dysenteriae*, and *S. typhi* [26]. INPs, primarily the silver variety, are an effective tool against multidrug-resistant bacteria, as can be seen from Table 1.

**Figure 1:**
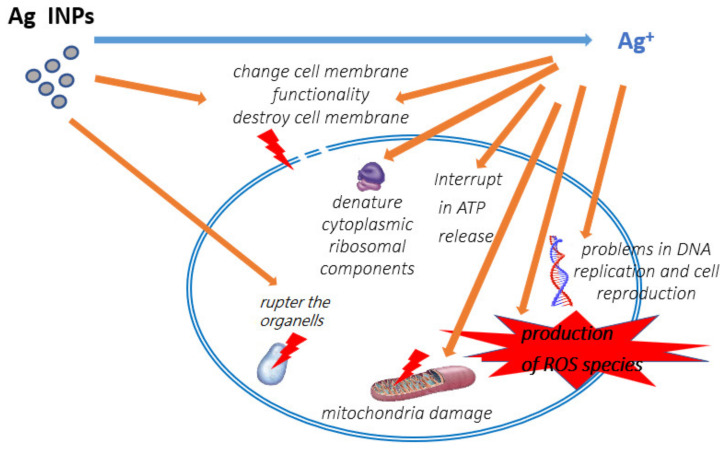
Supposed mechanisms of action of Ag INPs as antimicrobial agents.

The exact mechanism of silver NPs’ antibacterial action has not been entirely clarified. It is supposed that Ag INPs act on bacterial cells by different simultaneous mechanisms (Figure 1) [25,26]. Silver INPs can continually release silver ions, which can attach to cell walls due to electrostatic attraction and affinity to sulfur proteins [42]. The adhered ions can change the functionality and increase the permeability of the cytoplasmic membrane, and lead to disruption of the bacterial cell [43,44]. After the penetration of silver ions into the cell, the respiratory enzymes are deactivated, leading to the production of reactive oxygen species (ROS) and interruption in the adenosine triphosphate (ATP) release. Silver ions may interact with the sulfur and phosphorus components of DNA, which leads to damage the DNA replication and bacterial cell reproduction, or even results in termination of the microorganisms. Additionally, the Ag^+^ ions could efficiently hinder the protein synthesis by denaturing cytoplasmic ribosomal components [45,46,47].

Ag INPs may themselves destroy bacteria. Silver INPs smaller than 10 nm can directly alter cell permeability, enter bacterial cells, and cause their damage. The organelles might be ruptured due to denaturation of cell membrane, and cell lysis may occur. Additionally, Ag NPs can be injected into the microbial signal transduction pathway, which is impacted by phosphorylation of protein substrates. Here, Ag NPs can potentially dephosphorylate the tyrosine residuals over the peptide substrates [44,45,46].

Gram-negative bacteria are more susceptible to silver INPs. Their cellular wall is thinner than that of Gram-positive strains. The thick cellular wall may reduce the penetration of NPs into cells. The different antibacterial effects of silver INPs on Gram-negative and Gram-positive bacteria suggest that uptake of silver INPs is important to the antibacterial effect [45,46].

Since the size and shape of the Ag INPs influence the production of Ag^+^ (this phenomenon is described by the Ostwald–Freundlich equation), these factors should affect their antibacterial activity. Capping should also be taken into account in this connection. Because Ag NPs are small in size and sphere-shaped or quasi-spherical, they look like most suitable antibacterial agents. Zheng et al. found a significant increase in antibacterial activity, especially with Ag NPs smaller than 10 nm [47]. However, the study’s analysis of the effectiveness of triangular, spherical, rod-shaped, and truncated Ag INPs against *E. coli* in solution and agar plates showed the highest activity in triangular Ag INPs. This was followed by spheres and finally rods. This indicates that the mechanism of action of Ag-INPs is associated not only with ions.

Ag NPs can be applied to prevent multiple viral infections [48]. Spherical Ag INPs showed an inhibitory effect on adenovirus type 3 in an in vitro experiment [49]. The viral fluorescence intensity in adenovirus type-3-infected HeLa cells was reduced. Disruption of the adenovirus type 3 structure and viral DNA damage are possible causes of this inhibitory effect.

Ag NPs with average diameters of 10 nm and 50 nm inhibit hepatitis B virus genome replication [50]. Influenza-A-virus-infected MDCK cell cultures were used in another experiment with 5–20 nm Ag NPs without coating. It was established that Ag NPs were able to destroy the viral membrane glycoproteins that the virus uses to infect host cells [51].

Silver AgNPs also demonstrate broad-spectrum virucidal activity by preventing the interaction of human immunodeficiency virus-1 gp120 and cellular CD4, thereby inhibiting fusion or entry of the virus into the host cell [51].

Influenza-virus-infected MDCK cell cultures were used in another experiment to investigate the inhibitory potential of AgNPs. AgNPs (5–20 nm) with no coating were able to destroy the viral membrane glycoproteins that the virus uses to infect host cells [52].

AgNPs 10 nm diameter in size can reduce norovirus activity. The size of FCV (27–40 nm) is comparable to that of 10-nanometer AgNPs, and the interaction that occurs might be due to the similar sizes of the NPs and virus [53]. Another study reported a reduction in FCV VP1 viral capsid protein level in response to treatment with 10-nanometer AgNPs [54]. NPs are also able to inhibit poliovirus, zika virus, and human immunodeficiency virus type 1 [55].

Ag NPs also show antifungal activity against *C. albicans*, *C. tropicalis* [56,57], and *Fusarium oxysporum f. sp. radicis-lycopersici* [58]. The excellent antifungal activity of silver INPs against *C. albicans* is manifests in the destruction of cell membranes and suppression of the normal process of cell division [59]. The antifungal activity of silver INPs results from the formation of insoluble compounds with sulfhydryl groups in the cell walls of fungi and the disruption of the membrane-bound enzymes and lipids that cause cell lysis [60,61].

Thus, the antiviral and antifungal effects of silver INPs, as well their antibacterial activity, are based on various mechanisms of action, from membrane destruction to disruption of DNA function.

For other INPs, a similar pattern to that of Ag INPs has been observed. In recent decades, the number of scientific investigations devoted to their antibacterial, antiviral, and antifungal activity has increased, and they are assumed to have mechanisms of action similar to those of silver INPs, but there are still relatively few publications dedicated to their characterization. We focus on the main INPs below.

### 2.2. Cu, Cu_2_O, and CuO INPs

Copper and copper oxide INPs show the optimal ratio of the cost of precursors and antibacterial activity. Copper and its oxides are combined because a significant number of the methods for copper NPs synthesis do not allow researchers to obtain Cu INPs without oxide impurities, or cannot prevent their further oxidation [62].

Partially oxidized Cu INPs (Cu/Cu_2_O) with a crystallite size of 15 nm, according XRD analysis, were compared with silver INPs similar in size and synthesis method for their antibacterial activity against *Staphylococcus aureus* and *Escherichia coli* [63]. Cu INP antibacterial action was comparable with that of silver INPs against *S. aureus*, but showed slightly less antibacterial action against *E. coli*. The antibacterial activity of zerovalent copper NPs 12 nm in size against the Gram-negative bacterium *Escherichia coli* was assessed [64] in liquid as well as solid growth media. Copper INPs in concentrations of 60 µg/mL demonstrated complete cytotoxicity against *E. coli*. Their mechanism of antibacterial action is attributed to the adsorption of copper ions onto bacterial cells followed by the destruction of the bacterial cell wall. Cu_2_O NPs with 30 nm in size fabricated in reverse micellar templates exhibited antibacterial activity against Gram-positive *B. subtilis* and Gram-negative *P. aeruginosa* strains. Studies have been carried out based on cell viability, zone of inhibition, and minimal inhibitory concentration (MIC) values [65]. The MIC value was 62.5 µg/mL at pH5, and median inhibition concentration was 21.21 μg/L and 18.65 μg/mL for *P. aeruginosa* and *B. subtilis*, respectively. Cu INPs showed antimicrobial activity toward *Escherichia coli*, *Staphylococcus aureus*, and *Candida albicans* [66]. The mechanism of antibacterial action of Cu-NPs on *E. coli* cells was comprehensively studied by [67]. The results demonstrated that Cu NPs destabilize cell membranes. However, this was not the only reason for cell damage; Cu NPs cause multiple toxic effects, such as generation of ROS, lipid peroxidation, protein oxidation, and DNA degradation in *E. coli* cells [23]. CuO NPs in suspension were active against a range of bacterial pathogens, including *S. aureus*, meticillin-resistant *S. aureus*, *Staphylococcus epidermidis*, *E. coli*, and *Pseudomonas aeruginosa* [68]. CuO NPs are also active against *Escherichia coli*, *Klebsiella pneumoniae*, *Pseudomonas sps.*, *Proteus mirabilis* [39], methicillin resistant *Staphylococcus aureus*, and other drug-resistant bacterial strains (Table 1). The dependence of antibacterial activity on the release of ions from a metal surface was shown in [69] by means of the bioluminescent bacteria method. The authors used two recombinant *Escherichia coli* strains, one expressing bioluminescence constitutively and applicable for general antimicrobial testing, and the other induced by Cu ions.

The antiviral activity of Cu and CuO INPs has been less widely explored than Ag NPs. The antiviral application of CuO INPs has been evaluated against hepatitis C, herpes simplex virus, and Newcastle viral disease [70]. Copper NPs pose antifungal activity against *Fusarium* sp. [71], *Fusarium oxysporum*, *Phytophthora capsici* [72], *Candida albicans*, *Candida glabrata* and *Candida tropicalis* [73].

### 2.3. Au INPs

Au INPs are distinguished by properties significantly distinct from those of bulk materials. Therefore, they have promising potential for application in the fields of chemistry, physics, biology, and medicine. The biomedical applications of gold INPs include their implementation as carriers in targeted delivery systems, as basic components in biosensors, and as components in antibacterial HNC preparations.

Au INPs actively suppress bacterial growth and are characterized by a wide spectrum of action. Bacterial strains that can be inhibited by gold NPs include *S. epidermidis*, *S. aureus*, *E. coli*, *Bacillus megaterium*, *Bacillus subtilis*, *L. monocytogenes*, *S. typhimurium*, *K. pneumoniae*, *P. aeruginosa*, *B. subtilis* [74], *S. saprophyticus*, *E. faecalis*, *E. faecium*, *P. syringae*, *S. pyogenes*, *E. cloacae* [75], *Mycobacterium smegmatis*, *Mycobacterium tuberculosis* [76], and some antibiotic-resistant bacterial strains (Table 1). Au INP antibacterial activity increases with an increase in their specific surface area, as is typical for INPs. Their MIC concentrations for *S. mutans*, *S. salivarius*, and *S. sanguinis* decreased as the particle size lowered from 90 to 25 nm. [77]. Shape-dependent Au INP antibacterial activity was investigated with regard to *Escherichia coli*, *Pseudomonas aeruginosa*, and *Staphylococcus aureus* at lower concentrations [78]. Three types of AuNPs were used: nanospheres, nanostars, and nanocubes. Zones of inhibition of the bacteria strains (ZOI) values decreased in the sequence ZOI nanocubes> ZOI nanospheres> ZOI nanostars. The SEM images for *Escherichia coli*, *Pseudomonas aeruginosa*, and *Staphylococcus aureus* (treated with Au nanocubes, nanostars, and nanocubes) showed visible surface damage, disruptions and cell loss. Au nanostars formed larger aggregates and induced less surface damage. Au nanocubes exhibited complete surface damage and cell loss. The *S. aureus* cells were less susceptible to cellular damage as compared to the *P. aeruginosa* and *E. coli.*

Au NPs also exhibit antiviral activity (for instance, with measles virus [79], influenza A virus, [80], herpes simplex virus, and human immunodeficiency virus [81]). Gold INPs exhibit excellent antifungal activity against the fungus *Candida* [82]. Gold nanodiscs with much higher surface area displayed stronger fungicidal activity compared to gold polyhedral INPs.

### 2.4. ZnO INPs

ZnO INPs have advantages similar to those of copper-containing INPs: high antibacterial effectiveness at low concentrations (0.16–5.00 mmol/L), activity against a wide range of strains, and relatively low cost.

The two main antibacterial mechanisms proposed for ZnO-NPs are the release of zinc ions and ROS production [83,84,85]. The released Zn^2+^ has a significant effect on the active transport inhibition as well as on the amino acid metabolism and enzyme system disruption. Several studies have concluded that the Zn^2+^ ions exude into the growth media was responsible for the ZnO nanotoxicity, and the dissolution of ZnO-NPs forming Zn^2+^ was considered size dependent [86,87,88]. Therefore, engineered nanostructures might modify their toxicity by manipulating the dissolution rate.

Hydrogen peroxide (H_2_O_2_) generated from the ZnO powder slurry was detected using the oxygen electrode, and it correlated with the data of chemiluminescence analysis. The oxygen electrode may detect the generation of active oxygen from ZnO powder. Active oxygen species generated from the powder were associated with their antibacterial activities [89]. Several studies indicated ROS formation as the main mechanism responsible for the antibacterial activity of ZnO-NPs. Another mechanism of ZnO INP action is membrane disruption [90].

ZnO INPs poses bactericidal activity against *P. aeruginosa*, *E. coli*, *S. aureus*, *S. enterica*, *C. jejuni*, and *V. cholerae*, antiviral activity against Herpes Simplex Virus Type 1 and hepatitis A virus, and antifungal activity against *Botrytis cinerea*, *Penicillium expansum*, *Erythricium salmonicolor*, and *Candida albicans* [91,92,93,94,95,96,97]. ZnO nanoparticles in different ratios inhibited the growth of antibiotic-resistant *Mycobacterium tuberculosis* strains, but did not lead to bacterial death [98].

Particle size and concentration play important roles in the antibacterial activity of ZnO-NPs. ZnO-NPs of smaller sizes can easily penetrate into bacterial membranes due to their large interfacial area, which enhances their antibacterial efficiency [99]. ZnO INP antibacterial activity (*Staphylococcus aureus*, *Escherichia coli*) increased with decreasing particle size [100]. Nanopowders with an average particle size ranging from 0.1 to 0.8 μm were used. The influence of nanopowder particle size in activity against *Staphylococcus aureus* was less than that in activity against *Escherichia coli*. An increase in antibacterial activity against *Escherichia coli* with a decrease in the size of zinc oxide nanoparticles was also revealed in [101].

The bactericidal efficiency of ZnO against *Escherichia coli* was higher for the 12 nm INPs than for the 45 nm and 2 μm INPs. [102] The authors attribute the increased bactericidal action with the generation of H_2_O_2_ on the surface area of ZnO. A decrease in the particle size (an increase in the specific surface area value) leads to an increase in the number of oxygen species on the surface and to a higher antibacterial activity for the smaller nanoparticles.

### 2.5. TiO_2_ INPs

Titanium oxideTiO_2-_ INPs are also considered as an active component, not only in antibacterial systems, but also in anti-cancer systems, due to their excellent photocatalytic effect [103,104,105]. Their high antibacterial efficiency is connected with photodynamic antibacterial (PDA) and photothermal antibacterial (PTA) phototherapy. PDA phototherapy is based on the principle that photocatalytic materials can generate ROS under visible or near-infrared (NIR) light to oxidize phospholipids and proteins of bacteria, while an excess amount of ROS may cause cytotoxic effects. PTA phototherapy relies on photothermal agents that convert NIR light into local heating to kill bacteria.

TiO_2_ INP photocatalytic antibacterial activity has been shown for *E. coli*, *Pseudomonas aeruginosa*, *S. aureus*, *Enterococcus hirae*, and *Bacteroides fragilis* bacteria strains [106]. The photocatalytic antibacterial activity of TiO_2_ is attributed to the production of ROS, such as free hydroxyl radicals and peroxide [23]. The surface of TiO_2_ reacts with water by photocatalysis and releases hydroxyl radicals, which subsequently form superoxide radicals. The ROS synergistically act on phospholipids (polyunsaturated) on the surface of bacteria, causing site-specific DNA damage [23]. Due to its photocatalytic bactericidal effectiveness, TiO_2 -_INP_s_ is often used as a component of dental composites [107].

### 2.6. Other Metal INPs

Nine pure metals, namely Co, Ni, Zn, Zr, Mo, Ti, Sn, and Pb, were tested for their antibacterial properties against *Staphylococcus aureus* and *Escherichia coli*. Two assay methods were used: the film contact method and the shaking flask method. The film contact method produced better results than the shaking flask method, due to the more effective metal–microbe interaction occurring at the interphase. Co, Ni, Zn, Zr, Mo, and Pb were found to have antibacterial properties against both Gram-positive and Gram-negative bacteria strains. Therefore, they seem to be good candidates for application in antibacterial materials [108]. Al INPs showed an inhibitory effect on *Escherichia coli*, but only at high concentrations (1000 microg/mL [109]. Bi INPs showed good antibacterial activity against *H. pylori* strains. The obtained [41] MIC values varied between 60 and 100 μg/mL.

### 2.7. Metal Oxide INPs

Metal oxide INPs, such as MgO and CaO, are also under consideration as antibacterial agents. MgO INPs exhibit antibacterial activity against Gram-positive and Gram-negative bacteria, such as *S. aureus* (MIC; 1000 μg/mL), *E. coli* (MIC;500 μg/mL), *P. aeruginosa* (MIC; 1000 μg/mL), *Bacillus megaterium*, and *B. subtilis*. CaO INPs showed antimicrobial and antifungal activity against *Pseudomonas aeruginosa Staphylococcus epidermidis* and *Candida tropicalis* [110]. The MIC value of CaO NPs was found within the range of 2–8 mM for all the above-mentioned strains.

### 2.8. Magnetic INPs

Magnetic INPs are associated with a significant number of modern actively developing medical innovations, such as magnetic hyperthermia and MRI agents, magnetic separation, magnetic hyperthermia, and tissue engineering. They are considered as magnetic carriers and vectors in targeted drug delivery systems. The surface of the magnetic particle can be easily conjugated with the medicinal substance. The magnetic field not only enables retention of the drug composites in the target tissue, but also promotes (due to heating under the action of the magnetic field) the release of the drug molecule. In such targeted delivery systems, as can be seen from Table 2, magnetic INPs can also serve as antibacterial components. Potential magnetic antibacterial compositions include INPs of zinc ferrite, which is a mixed oxide of iron and zinc, and INPs composed of superparamagnetic oxides of iron and ferrites.

## 3. Complex INPs

The production of particles with an optimal ratio of magnetic properties and antibacterial activity has seen an increasing trend in recent years. Among such developments, it has been found that the superparamagnetic iron oxides magnetite and maghemite cobalt, along with nickel ferrites, can be modified to form complex particles, while iron oxides or Co or Ni ferrites can be diluted with zinc and copper ferrites, as well as AG-INPs (Table 2).

In Ag@ZnFe_2_O_4_ complex NPs with different weight ratios of AgNO_3_ (0, 0.25, 0.50, and 0.75 μg/mL) exhibited antibacterial activity against *S. aureus*, *S. sciuri*, *S. lentus*, *S. vitulinus*, and *E. columbae* [125]. The MIC concentrations of the HNCs depended on the ratio of components and on the bacterial strain, and ranged from 0.4 to 1.5 μg/mL, which is significantly lower than the values of zinc ferrite (6–25 μg/mL). Unfortunately, the authors did not compare the activity of the systems with the activity of silver INPs. However, the MIC values of hybrid particles are lower than the values given for silver INPs in Table 1.

According to the qualitative disk-diffusion method results Ag@CoFe_2_O_4_ NPs exhibited an improved inhibitory effect against the bacteria and fungi tested in [126,127]: *Escherichia coli*, *Pseudomonas aeruginosa*, *Staphylococcus aureus*, *Bacillus subtilis*, and *Candida albicans*. The quantitative assay of the Ag@CoFe_2_O_4_ antimicrobial activity exhibited MIC values ranging from 0.031 to 0.062 mg/mL against all tested bacterial strains. The MIC value for cobalt ferrite was significantly higher, and varied from 0.125 to 1 μg/mL. Hybrid particles retained the superparamagnetic behavior required to control their systems through magnetic fields.

A combined influence on properties is considered not only for hybrid inorganic particles with magnetic and bactericidal properties, but also for two antibacterial components.

A synergistic effect resulting in an increase of antibacterial activity was found to be characteristic of combinations of metal INPs and their oxides with antibiotics. Ag-ZnO*mSiO_2_ HNCs were tested on both Gram-positive and Gram-negative bacterial strains (*E. coli*, *Pseudomonas aeruginosa*, *Streptococcus salivarius*, and *Stafilococcus aureus*) and *Candida albicans*. A positive synergistic antimicrobial effect was revealed for Ag and Zn agents [128].

Ag and Cu INPs in combination against *S. aureus* and *P. aeruginosa* also significantly increase their antimicrobial effects compared to single components [129]. Thus, certain metallic INPs can be used in combination to improve antimicrobial efficiency. Compared to ZnO nanoparticles, ZnO-Mn nanoparticles have higher antimicrobial activity against *K. pneumoniae*, *Shigella dysenteriae*, *S. enterica Typhimurium*, and *P. aeruginosa* [130].

Cu-doped ZnO INPs were tested for antibacterial activity against Gram-positive (*Staphylococcus aureus*, *Streptococcus pyogenes*) and Gram-negative (*Escherichia coli*, *Klebsiella pneumonia*) bacteria via agar disk-diffusion method. The results showed that Cu-doped ZnO-NPs exhibit higher antibacterial activity towards Gram-positive bacteria than towards Gram-negative bacteria. It has been observed that Gram-positive microbes are more susceptible to Cu-doped ZnO INPs as compared to Gram-negative microbes. The growth-inhibition zones were larger in the case of hybrid particles than in the case of zinc oxide [131].

TiO_2_/ZnO nanoparticles have a more pronounced bactericidal effect against E. coli compared to ZnO nanoparticles, while Ag/TiO_2_/ZnO nanoparticles are more effective than TiO_2_/ZnO nanoparticles [132].

The antibacterial activity of reduced graphene oxide—nAg HNCs against several important human pathogenic multi-drug-resistant bacteria, namely Gram-positive coccal Staphylococcus aureus and Gram-negative rod-shaped Escherichia coli and Proteus mirabilis were investigated [133]. At the same concentration (100 µg/mL), graphene oxide-nAg HNCs was significantly more effective against all three pathogens than either graphene oxide or nAg. The increase in antibacterial activity in the case of HNCs was attributed by the authors to the physical interaction between the sharp edges of graphene oxide sheets, disrupting the cell membrane and facilitating the transport of silver ions across the cell membrane.

Nowadays there are only few studies of the antibacterial activity of the two antibacterial components. However, preliminary results open the prospects for the creation of complex inorganic antibacterial systems that will allow the combination, and in some cases, the improvement of the properties of individual components.

## 4. Nanocomposites of INPs with Antibacterial Drug Substances

### 4.1. Drug Carriers

**Figure 2:**
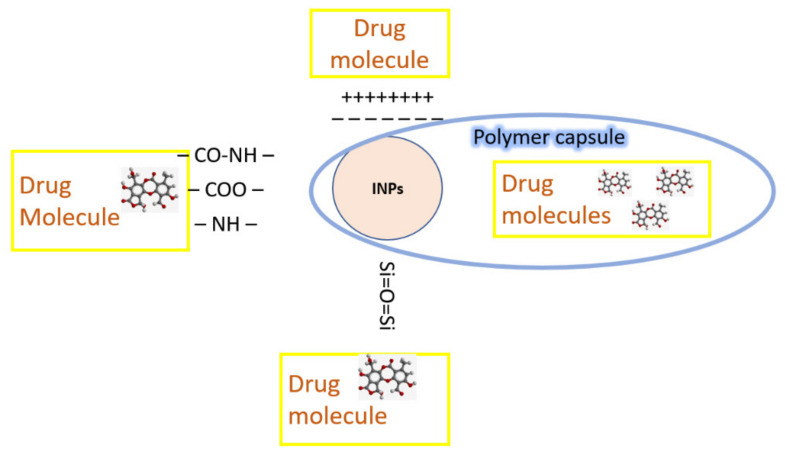
Drug conjugation with INPs.

Targeted drug delivery is a rapidly developing biomedical area associated with INPs. In this direction, INPs are considered as potential carriers and magnetic vectors (in the case of magnetic NPs) that enable the magnetic field to keep the drug preparation within the target tissue. Metal ions from the surfaces of INPs are thought to form tight coordination bonds with the N, O, and S atoms in biomolecules, as well as amines, phosphates, protein thiols, and DNA. It is also possible to bind INPs with drug molecules by electrostatic interaction, encapsulating the molecules and INPs into polymer particles or fixing them onto the surfaces of silica gels [134] (Figure 2).

Among metals and their oxides, Au INPs are most often considered as carriers, due to their non-toxic and non-immunogenic properties [135]. In addition, Au-INPs are characterized by their easily formation NPs of various shapes, which can be easily conjugated with drug molecules [136]. Due to their sensitivity and selectivity response to the biological environment, the optical properties of Au INPs have also been used in sensing biological molecules and cells. The advantages of gold INPs as biosensors include their ability to change the position of the plasmon resonance signal by changing the shape and size of the particles [137]. A significant disadvantage of these carriers is their high cost.

Other promising drug carriers are the magnetic INPs of magnetite, maghemite, ferrites. These NPs, primarily iron oxide INPs, are characterized by relatively low cost, high biocompatibility, and biodegradability [138]. Their prospects as agents for MRI, as well as the optical properties in the case of gold INPs, make it possible to use magnetic particles not only in therapeutic (drug carrier) applications, but also in diagnostic field [139]. For these particles it is possible to promote the release of the drug molecule by heating them in a magnetic field. Magnetic INPs could be chemically fictionized with different drug molecules of different strategies [140], such as noncovalent drug conjugation (drug adsorption via electrostatic/hydrophobic interactions, drug loading via encapsulation), covalent drug conjugation, and hybrid drug-conjugation. Hybrid drug-conjugated systems combine both covalent and noncovalent mechanisms for stimulus-controlled drug release.

### 4.2. INPs as Components of Hybrid Nanocomposites (HNCs)

Besides having excellent drug-carrier properties, INPs can also improve the antibacterial effects of loaded antibacterial drugs by expanding the spectrum of bactericidal action, since INPs are active against antibiotic-resistant bacterial strains. In addition, for several of antibacterial drugs and metal INPs, a synergistic effect increasing their antibacterial activity as a result of various combinations has been established in the last decades.

In one study, the strong synergistic efficiency of multiple antibiotics (piperacillin, piperacillin, aztreonam, meropenem, cefoperazone, ceftazidime, cefepime, gentamicin, amikacin, ofloxacin, and colistin) with different chemical structures and modes of action in combination with Ag INPs (of an average size of 28 nm with a narrow size distribution) against *Escherichia coli*, *Pseudomonas aeruginosa*, and *Staphylococcus aureus* was determined [141]. The microdilution method of determining the MICs of antibiotics was used. The antibacterial activity of the tested antibiotics increased markedly when combined with Ag INPs (even at low concentrations).

Ag INPs improve the sensitivity of *E. coli* bacteria to gentamicin, kanamycin, geneticin, and tetracycline [142]. Additionally, five clinically multi-drug-resistant Gram-negative pathogen strains, namely *Acinetobacter baumannii*, *Enterobacter coli*, *Klebsiella pneumonia*, and *Pseudomonas aeruginosa*, were isolated and incubated with serial concentrations of Ag^+^ and antibiotics (gentamycin; ranamycin; geneticin; tetracycline; ebselen) in combination for 24 h. The results showed that Ag^+^ and antibiotics in combination might be the only effective weapon against a range of resistant bacteria. The synergistic antibacterial effect of Ag and antibiotics was correlated with the production of ROS.

Synergetic antibacterial effects were observed in most combinations of Ag NPs, chitosan, and antibiotics (azithromycin, levofloxacin, and tetracycline). Two Gram-negative bacteria, *Escherichia coli* and *Klebsiella pneumonia*, and two Gram-positive strains, *Staphylococcus aureus* and *Enterococcus faecalis*, were used. The MIC of the drugs was reduced, and the drug efficiency increased from 37 to 97% [143].

Synergetic antibacterial activity against *E. coli*, *S. typhi*, and *M. luteus* was observed for copper INPs with ampicillin, amoxicillin, gentamicin, and ciprofloxacilin [144]. It was proposed that active groups in the antibiotic molecule, such as hydroxyl and amido, reacted with the nanosized metallic copper by chelation, and the reaction led to synergism. Synergistic antibacterial effects were observed for silver and copper HNCs with the antibiotics tetracycline and kanamycin against the biorecycling microbes *Bacillus subtilis* and *Pseudomonas fluorescens* [145]. A group of antibiotics (azithromycin, cefotaxime, cefuroxime, fosfomycin, and chloramphenicol) had a synergistic effect in the presence of ZnO and Ag INPs against *Escherichia coli*.

The antimicrobial activities of silver and zinc oxide INPs, alone and in combination with antibiotics, were examined against strains of pathogenic microorganisms, such as *Staphylococcus aureus*, *Salmonella enterica subsp. Bukuru*, *Escherichia coli*, and *Candida albicans* by normal disk-diffusion method. The synergistic effect of antibiotics against E. coli was significantly increased in the presence of Ag INPs compared to antibiotics without Ag INPs.

Synergistic and additive antibacterial effects were detected in combinations of ZnO INPs with selected antibiotics (meropenem trihydrate, ciprofloxacin, and colistin sulfate) [146,147]. Experiments were carried out with *E. coli* and *A. baumannii* bacterial strains from clinical samples. The efficacy of vancomycin was improved in combination with ZnO (20 nm) nanoparticles. The results showed a decrease in vancomycin MICs from (2500–5000) µg/mL to (39–78.125) μg/mL when it was mixed with ZnO 20 nm. The study was carried out on various clinical samples collected from patients attending Al Salam and Al-Khansa Hospitals [148]. However, the antibacterial action of the combination of Ag INPs with oxacillin and neomycin against *Staphylococcus aureus* decreased in comparison with the action of antibiotics only [147]. Ampicillin-functionalized Au and Ag INPs produces a combined antibacterial effect that can destroy ampicillin-resistant bacteria [136]. Cryoformed HNCs of dioxidine and gentamicin combined with Ag and Cu INPs showed an increase in antibacterial activity compared to the separate activity of the individual components [144,149,150].

Information on the presence of a synergistic effect resulting from the combination of INPs composed of metals and their oxides with antibiotics is summarized in Table 3. Thus, it can be concluded that due to the combination of metal INPs and antibiotics, it is possible to broaden not only the spectrum of bactericidal action, but also to increase the antibacterial effectiveness of hybrid targeted drug delivery systems based.

## 5. Concluding Remarks and Perspectives

**Figure 3:**
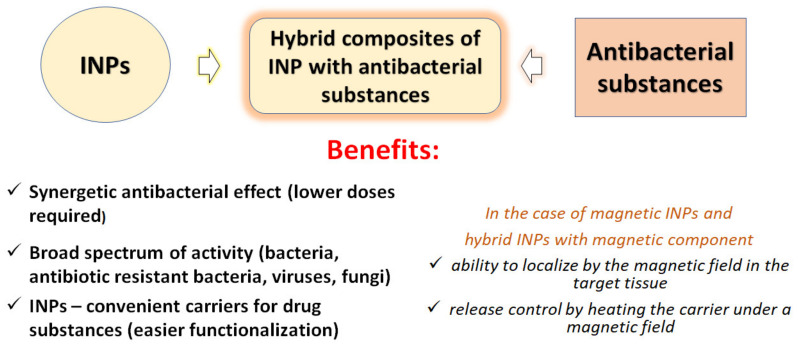
Benefits of INPs as components of new-generation antibacterial preparations.

The use of antibiotics in medicine has saved the health and lives of a great number of patients with bacterial infections, and it has made it possible to cure many previously incurable diseases. However, the growing emergence of bacterial strains resistant to antibiotics has resulted in the appearance of bacterial diseases that cannot be treated. This is one of the key problems requiring an urgent solution at the present time. It is necessary to develop a new generation of antibacterial agents that are active against pathogenic microorganisms, including resistant ones. For the last 20 years, metal INPs have been considered as such potential agents, primarily those of metals with a pronounced bactericidal effect, such as silver, gold, copper, and zinc oxide [134]. Their benefits are summarized on Figure 3. It was found that due to their mechanisms of action on the bacterial cell, which are different from the mechanisms of action of antibiotics, they are able to suppress pathogenic antibiotic-resistant microorganisms. They are characterized by high antibacterial activity and a wide spectrum of action, including not only bacteria, but also fungi and viruses.

However, INPs have significant potential not only as effective antibacterial components of drugs, but also as possible carriers of next-generation drugs providing targeted delivery and controlled release into diseased tissue. When studying the antibacterial properties of such hybrid systems of antibacterial drugs, it emerged that they are characterized not only by their expansion of the spectrum of action, but also by the mutual enhancement of the antibacterial activities of INPs and antibiotics [130,131,132,133,134,135,136,137,138,139,140,141].

Currently, there are works devoted to the optimization of such nanocomposites of INPs carriers-antibacterial agents. It emerges that the synergistic effect is characteristic not only of INPs combined with antibiotics, but also of hybrid combinations of different INPs. A decrease in the concentration of expensive components, namely gold and silver, made it possible to reduce the cost of future HNCs incorporating antibacterial drugs. In addition, another trend that has just begun to develop is the production of mixed or hybrid carrier INPs containing an active bactericidal metal component (gold, silver, copper, or zinc oxide) and magnetic INPs. Coating the magnetic particle cores with an antibacterial shell, or obtaining mixed cobalt ferrites with copper and zinc, are among the trends.

The magnetic components of the particles become magnetic vectors and allow control of the drug particle location using a magnetic field. In addition, heating the particle in a magnetic field makes it possible to influence on the release of the drug component [4,5,6].

Before therapeutic use, it is necessary to confirm that the effective concentrations (in terms of antibacterial and magnetic properties) of inorganic nanoparticles are non-toxic to the human body. There is still a range of clinical and preclinical trials to be conducted. However, the primary task currently facing researchers is the establishment of new applications for complex INPs and their HNCs including drug substanc4es.

## Figures and Tables

**Table 1:** Research addressing the use of INPs against drug-resistant bacteria.

NPs, Size	Bacteria Tested (Drug to Which It Is Resistant)	Method and Concentrations	Reference
Ag 5–20 nm	*Staphylococcus aureus* (methicillin)	MIC: 32 μg/mL	[33]
Ag -	*P. aeruginosa* (ampicillin, nitrofurantoin, nalidixic acid, ciprofloxacin)	MIC: 12.5 μg/mL	[34]
Ag 45 nm	*E. coli* (erythromycin, amoxicillin, tetracycline, and streptomycin) *L. monocytogenes* (rifampicin, cefotaxime, tetracycline, gentamycin, and chloramphenicol) *S. typhimurium* (norfloxacin, amoxicillin, ciprofloxacin, chloramphenicol, and trimethoprim/sulphamethoxazole) *P. aeruginosa* (ciprofloxacin, norfloxacin, streptomycin, and levofloxacin.) *K. pneumoniae* (neomycin, kanamycin, tetracycline, nalidixic acid, amoxicillin, and gentamycin)	MIC: *E. coli* 6.25 μg/mL *L. monocytogenes* 12.5 μg/mL *S. typhimurium* 3.125 μg/mL *P. aeruginosa* 6.25 μg/mL *K. pneumoniae* 25 μg/mL	[35]
Ag 20 nm	*E. coli*, *K. pneumoniae*, *S. aureus*, *P. aeruginosa* (tetracycline, ampicillin, and erythromycin)	Disk diffusion method, Ag NPs showed bacteriolytic activity at all tested concentrations: 10, 30, 60, 90, and 120 µg/µL	[36]
Ag 12 nm	*Pseudomonas aeruginosa* (amikacin, aztreonam, ceftizoxime, cefepime, gentamicin, imipenem, netilmicin, ofloxacin, piperacillin and tazobactam; the strains which were resistant to 6 or 7 antibiotics from the list above were used)	MIC: 6.25 μg/mL	[37]
CuO 62	*Staphylococcus aureus*, *Streptococcus mutans*, *Streptococcus pyogenes*, *Streptococcus viridans*, *Staphylococcus epidermidis*, *Corynebacterium xerosis*, and *Bacillus cereus*, *Escherichia coli*, *Klebsiella pneumonia*, *Pseudomonas aeruginosa*, *Proteus vulgaris* (multidrug-resistant clinical bacterial strains)	Disk diffusion method, concentration of CuO NRs 1.25 mg/50 µL DMSO	[38]
CuO 25–30 nm Fe_2_O_3_ 25–30 nm	*Staphylococcus aureus* (methicillin), *Staphylococcus epidermidis* (methicillin), *Enterococcus faecalis* (vancomycin)	Disk diffusion method, MIC 30–40 μg/mL	[39]
Au 25 nm	*Pseudomonas aeruginosa* and *Staphylococcus aureus* (azithromycin, chloramphenicol, tetracycline, nitrofurantoin, cefotaxime, amoxicillin, sulphamethoxazole, novobiocin, cephalothin, methicillin, bacitracin, ampicillin and aztreonam)	Disk diffusion method	[40]
Bi 1–5 nm	Helicobacter pylori (multiple-antibiotic)	MIC: 100 μg/mL	[41]

**Table 2:** Antibacterial activity of magnetic NPs against bacteria strains.

NPs	NPs Size	Bacteria Tested	Method and Concentrations	Reference
Fe_3_O_4_/γ-Fe_2_O_3_	10–20 nm	*Escherichia coli*, *Bacillus subtilis* (Relatively high ROS production was indicated in upon Fe_2_O_3_ treatment of the bacteria)	The BacLight fluorescence assay, bacterial growth kinetic and colony-forming unit studies, 2.5, 5, 10, 25, and 50 μM	[111]
Fe_2_O_3_	50–110 nm	*Staphylococcus aureus*, *Escherichia coli*	Disk diffusion method, 4 mg/mL	[112]
Fe_3_O_4_	14 nm	*Staphylococcus aureus*, *Proteus mirabilis*, *Pseudomonas aureginosa*	Disk diffusion method, 11.50–36.30 mg/mL	[113]
Fe_3_O_4_	10–100 nm	*E. coli*, *Staphylococcus aureus*, *Bacillus subtilis* and *Pseudomonas aeruginosa*	Disk diffusion method, 0.029–0.047 mg/mL	[114]
CoFe_2_O_4_	35 nm	*Staphylococcus aureus*, *Candida albicans and Rhizopus oryzae fungal strain*	MIC: 25 μg/mL	[115]
CoFe_2_O_4_	20–30 nm	*Pseudomonas aeruginosa*, *Escherichia coli*, *Staphylococcus aureus*, *Bacillus cereus*	MIC (μg/mL): *Pseudomonas aeruginosa* 0.24, *Escherichia coli* 0.12, *Staphylococcus aureus* 0.24, *Bacillus cereus* 0.06	[116]
Cu_1−x_NixFe_2_O_4_	20–60 nm	*Escherichia coli*, *Klebsiella pneumonia*, *Staphylococcus aureus*, *Bacillus subtilis*	Disk diffusion method	[117]
NiFe_2_O_4_	15–50 nm	*Escherichia coli*, *Staphylococcus aureus*	MIC: 5 mg/mL	[118]
Ni, Co, Fe, Zn ferrites		*Escherichia coli*, *Staphylococcus aureus*	Disk diffusion method	[119]
ZnFe_2_O_4_		*Staphylococcus aureus*, *Streptococcus Pneumoniae*, and carbapenem-resistant *Enterobacteriaceae*	Disk diffusion method, 12.5–100 mg/mL	[120]
Ag-decorated ZnFe_2_O_4_	25 nm	*Staphylococcus vitulinus*, *S. aureus*, *Enterococcus columbae*, *Staphylococcus lentus*	MIC: 0.4–1.5 μg/mL	[121]
ZnFe_2_O_4_, CoFe_2_O_4_, Zn_0.5_Co_0_._5_Fe_2_O_4_		*Escherichia coli*, *Staphylococcus au-reus*	IC50 (µg/mL): ZnFe_2_O_4_–460, CoFe_2_O_4_–980, Zn_0.5_Co_0_._5_Fe_2_O_4–_465	[122]
Ag@ZnO		*Staphylococcus aureus*	Noncytotoxic doses of Ag@ZnO stimulate proliferation and migration of human keratinocytes. Ag@ZnO increases the expression of antimicrobial peptides hBD2 and RNase7 and lysosomal degradation of intracellular bacteria.	[123]
Ag-CoFe_2_O_4_	23–29 nm	*E. coli*, *Pseudomonas aeruginosa*, *Staphylococcus aureus*, *Enterococcus faecalis*, *Candida albicans*	MIC: 2 μg/mL	[124]

**Table 3:** Publications devoted to the identification of the synergistic antibacterial effect of antibiotics with INPs against bacterial strains.

NPs	Antibiotic	Bacteria or Fungi Tested	Reference
Ag, Cu	Tetracycline, kanamycin	*Bacillus subtilis*, *Pseudomonas fluorescens*	[143]
Ag, Cu	Gentamicin, dioxidine	*S. aureus*, *E. coli*	[151,152]
Ag	Ciprofloxacin, streptomycin and gentamicin	*Staphylococcus aureus*, *Pseudomonas. Aeruginosa*, *Escherichia coli*	[153]
Ag	Azlocillin	*P. aeruginosa*	[154]
Ag	Erythromycin, ampicillin, chloramphenicol, cephalothin, clindamycin, tetracycline, gentamycin, amoxicillin, ciprofloxacin, cefpodoxime, cefuroxime	Multiresistant *S. aureus*, *S. mutans*, *S. oralis*, *S. gordonii*, *Enterococcus faecalis*, *E. coli*, *A. actinomycetemcomitans*, *P. aeruginosa*	[155,156,157]
Ag	Vancomycin, ampicillin, penicillin	*S. aureus*, *E. coli*, *K. pneumoniae*	[158]
Ag	Vancomycin, amikacin	*E. coli*, *S. aureus*	[159]
Au	Ampicillin	*S. aureus*	[160]
Au	Kanamycin	*Staphylococcus epidermidis*, *Streptococcus bovis*, *Enterobacter aerogenes*, *Pseudomonas aeruginosa*	[161]

## Data Availability

Not applicable.
